# Comparative Study of Different Sampling Methods of Biofilm Formed on Stainless-Steel Surfaces in a CDC Biofilm Reactor

**DOI:** 10.3389/fmicb.2022.892181

**Published:** 2022-06-13

**Authors:** Nissa Niboucha, Coralie Goetz, Laurie Sanschagrin, Juliette Fontenille, Ismaïl Fliss, Steve Labrie, Julie Jean

**Affiliations:** ^1^Département des Sciences des Aliments, Université Laval, Québec, QC, Canada; ^2^Institut sur la Nutrition et les Aliments Fonctionnels (INAF), Université Laval, Québec, QC, Canada

**Keywords:** biofilm sampling methods, CDC biofilm reactor, dairy processing plant, stainless-steel surfaces, sonication, synthetic sponge, *Pseudomonas azotoformans*

## Abstract

The formation of biofilms in dairy processing plants can reduce equipment efficiency, contribute to surface deterioration, and contaminate dairy products by releasing the microorganisms they contain, which may cause spoilage or disease. However, a more representative identification of microbial communities and physico-chemical characterization requires to detach and recover adequately the entire biofilm from the surface. The aim of this study is to develop an efficient technique for in-plant biofilm sampling by growing a strain of *Pseudomonas azotoformans* PFl1A on stainless-steel surface in a dynamic CDC biofilm reactor system using tryptic soy broth (TSB) and milk as growth media. Different techniques, namely, swabbing, scraping, sonic brushing, synthetic sponge, and sonicating synthetic sponge were used and the results were compared to a standard ASTM International method using ultrasonication. Their efficiencies were evaluated by cells enumeration and scanning electron microscopy. The maximum total viable counts of 8.65 ± 0.06, 8.75 ± 0.08, and 8.71 ± 0.09 log CFU/cm^2^ were obtained in TSB medium using scraping, synthetic sponge, and sonicating synthetic sponge, respectively, which showed no statistically significant differences with the standard method, ultrasonication (8.74 ± 0.02 log CFU/cm^2^). However, a significantly (*p* < 0.05) lower cell recovery of 8.57 ± 0.10 and 8.60 ± 0.00 log CFU/cm^2^ compared to ultrasonication were achieved for swabbing and sonic brushing, respectively. Furthermore, scanning electron microscopy showed an effective removal of biofilms by sonic brushing, synthetic sponge, and sonicating synthetic sponge; However, only the latter two methods guaranteed a superior release of bacterial biofilm into suspension. Nevertheless, a combination of sonication and synthetic sponge ensured dislodging of sessile cells from surface crevices. The results suggest that a sonicating synthetic sponge could be a promising method for biofilm recovery in processing plants, which can be practically used in the dairy industries as an alternative to ultrasonication.

## Introduction

Biofilms are complex and dynamic communities of microorganisms that grow on solid surfaces. They are a major concern in the dairy industry, where they may develop on processing equipment and become an inveterate source of constant contamination of products. Many microorganisms in biofilms synthesize extracellular polymeric substances (EPS) that envelope them in a highly hydrated matrix, thereby enabling them to persist in harsh environments (Boltz et al., 2017). Beside polysaccharides, other substances including proteins, lipids, and extracellular DNA (eDNA) all may contribute to form the matrix, thus promoting cell-cell and cell-substrate interactions and helping to create mechanical stability and strong network structure in the biofilm (Flemming and Wingender, 2010).

The biofilm formation on processing plant surfaces decreases thermal efficiency, such as of heat exchangers, mainly due to lower heat transfer (Lelieveld et al., 2005; Mogha et al., 2014; Jindal et al., 2018). Moreover, biofilms may contribute to decrease the fluid flow within processing pipelines (Seale et al., 2015). Biofilms can also accelerate microbially-induced corrosion of stainless steel of which most processing equipment is made (Lee and Newman, 2003; Ramesh Babu et al., 2006; Gupta and Anand, 2018). In addition, the release of different microorganisms and their metabolites present in the biofilms, may greatly affect the safety and quality of milk and other dairy products, thus resulting in serious economic losses (Teh et al., 2014; Parkar et al., 2015; Seale et al., 2015).

Among the options currently available to suppress biofilm formation in dairy plants, conventional cleaning-in-place (CIP) procedures consisting of treating food processing surfaces with cleaning and sanitizing solutions are by far the most commonly adopted (Marriott et al., 2018). However, many bacteria found in biofilms are resistant to conventional sanitizers, mainly because of the protection provided by the biopolymer matrix of EPS (Renner and Weibel, 2011; Jindal et al., 2016). Therefore, the development of disinfectants that could effectively eradicate dairy biofilms requests complete characterization of both the bacteria and the matrix components involved, which requires adequate sampling techniques.

Several methods are commonly used for routine microbiological monitoring of food processing surfaces, namely direct agar contact, swabs, sterile cloths, and sponges (ISO 18593 ISO, 2018), depending on the shape and size of the surface. Although simple and convenient, these methods generally fail to effectively detach the biofilm from the surface, and the bacteria remain trapped in the polymer matrix (Bredholt et al., 1999). Consequently, these conventional methods provide a low recovery rate of biofilms. Thus, the resulting microbiological data are inevitably biased, and the microbial population may be underestimated or misrepresented, with a high risk of the presence of unidentified pathogens (Branck et al., 2017).

More rigorous mechanical methods of biofilm sampling include manual scraping using a spatula (Gunduz and Tuncel, 2006; Goeres et al., 2009; ASTM International, 2017; Leonetti et al., 2020) or using powered devices to generate vibrations such as ultrasound (Branck et al., 2017) or other high-shear hydrodynamic phenomena. For example, one standard method proposed by ASTM uses ultrasonication at 45 kHz to detach and sample *Pseudomonas aeruginosa* biofilms grown on stainless steel coupons (ASTM International, 2019). Although this method provides reproducible results, it is not practically applicable on industrial dairy equipment surfaces.

Recent reports have shown that sonication, used in sonic toothbrushes, appeared to be efficient for dislodging dental plaque biofilms (Singh et al., 2011; Schmidt et al., 2013; Re et al., 2015). In addition to the vigorous physical brushing motion of sonic toothbrush bristles, fluid dynamics may allow disruption and removal of biofilm even when the device is held 4 mm above the substrate surface (Heersink et al., 2003).

The objective of this study is to optimize a biofilm sampling technique using the biofilmogenic strain *Pseudomonas azotoformans* PFl1A, in a dynamic CDC biofilm reactor (CBR) system. The performance of five methods including swabbing, scraping, sonic brushing, synthetic sponge, and sonicating synthetic sponge, an in-house developed system, was evaluated and compared with ultrasonication, a standard method established by ASTM International. To determine which technique provided the most complete recovery of cells from biofilm, the stainless-steel surfaces were observed by scanning electron microscopy (SEM) after biofilm detachment.

## Materials and Methods

### Milk Skimming and Sterilization

Raw milk was provided by a local dairy plant (Agropur, Quebec, Canada). Twenty-two liters were used for each experiment with three repetitions. Upon delivery, the milk was skimmed using a DeLaval 619 cream separator (DeLaval, Peterborough, ON, Canada) then sterilized at 140°C for 4 s using a Microthermics UHT/HTST 25HV Hybrid Lab pasteurizer (Microthermics Inc., Raleigh, NC, United States).

### Culture Preparation

The strain of *Pseudomonas azotoformans* PFl1A, used in the present study, was isolated from a local dairy processing plant (Goetz et al., 2021) and stored at −80°C in Bacto*™* tryptic soy broth (TSB; BD Canada, Mississauga, Ontario) containing 20% (v/v) glycerol (Invitrogen, Thermo Fischer Scientific, CA, United States). One hundred microliters of thawed bacterial solution were added to 100 mL of TSB (300 mg/L). The culture was then incubated overnight at 30°C on a rotary shaker at 160 rpm to obtain a bacterial count of about 10^8^ CFU/mL. This inoculum was also prepared using 100 mL of sterile skimmed milk following the similar conditions as for TSB.

### Biofilm Formation Using the CDC Biofilm Reactor

Biofilms of *P. azotoformans* PFl1A were developed on eight stainless steel slides (316 grade, 76 mm × 15 mm) (CBR 2128-316, BioSurface Technologies Corporation, Bozeman, MN, United States) in the CDC biofilm reactor (CBR 90, BioSurfaces Technologies Corporation, Bozeman, MN, United States) according to the standard protocol (ASTM International, 2017). Briefly, the reactor was charged with 340 mL of TSB medium (300 mg/L) inoculated with 1 mL of overnight culture. Stirring was set at 130 rpm and the temperature was maintained at 30°C in a batch mode using a hotplate (VWR International, NJ, United States). After 24 h, TSB medium (100 mg/L) was fed by a peristaltic pump (HV-77913-70 Masterflex^®^, Cole-Parmer Company, Montreal, QC, Canada) to the reactor for 24 h in a continuous mode at a flow rate of 11.3 mL/min from a 20 L carboy placed upstream (Supplementary Figure 1). The effluent from the reactor was recovered simultaneously (by overflow) in the downstream carboy. The procedure using sterile skim milk was identical.

### Biofilm Sampling Methods

Before harvesting biofilm, each stainless-steel slide was rinsed three times by immersion in phosphate-buffered saline (PBS; 137 mM NaCl, 2.7 mM KCl, 8 mM Na_2_HPO_4_, 2 mM KH_2_PO_4_) to eliminate planktonic cells. Each method was tested on two slides and biofilm was recovered from both sides.

#### Ultrasonication

This technique was carried out according to the ASTM International method with slight modifications (ASTM International, 2019). Briefly, biofilm was harvested by vortexing the slides in 42 mL of PBS for 30 s at a maximal speed then sonicating at 40 kHz for another 30 s, using a Branson CPX2800H ultrasonic water bath (Branson Ultrasonics Corporation, Brookfield, CT, United States) at a power of 110 W. This operation was repeated 3 times to dislodge attached bacteria and disaggregate them to obtain a homogeneous cell solution.

#### Swabbing

Biofilm was removed by rubbing the slide surfaces with sterile 6′′ swabs (Puritan Medical Products Company, Guilford, ME, United States) soaked in PBS (one for each slide face). The cotton ends were detached from the swabs and placed in a sterile tube containing 42 mL of PBS (ISO 18593 ISO, 2018). This solution was vortexed (30 s) and sonicated (30 s) thrice as mentioned in the previous section.

#### Scraping

Biofilm was scraped from the slides using a sterile flat-edge Teflon spatula (Saint-Gobain Performance Plastics, Poestenkill, NY, United States) and suspended in 42 mL of sterile PBS. The suspension was vortexed and sonicated as described above.

#### Sonic Brushing

A Sonicare electric toothbrush (4100 Protective Clean, Philips) operating at a frequency of 257 Hz was cleaned thoroughly with a laboratory detergent, disinfected with 70% alcohol (v/v), air dried then sterilized by exposure to ultraviolet radiations (UV) for 1 h. For biofilm recovery, the toothbrush head was immersed in PBS then used immediately to brush the slide for 40 s inside a sterile container (to contain splashing). After sonic brushing, the toothbrush head was released into the container and 42 mL of PBS were added to rinse the scrubbed slide. The biofilm suspension obtained was vortexed and sonicated as described above.

#### Synthetic Sponge

The biofilm-covered slide was sampled with a wet EZ Reach*™* sponge (World Bioproducts, Libertyville, IL, United States) soaked in PBS. The surface was scrubbed firmly in one direction while applying a constant pressure over entire area of the slide. The sponge was then detached from its holder and placed in a stomacher bag containing 42 mL of PBS to suspend the biofilm, which was then homogenized in a Pulsifier^®^ (Microgen Bioproducts Ltd., Surrey, United Kingdom), for 30 s at a maximal force of 3,000 rpm. Each face of the sponge was used to remove biofilm from one side of the slide (ISO 18593 ISO, 2018).

#### Sonicating Synthetic Sponge

A sampling device was developed by our team combining the synthetic sponge with the vibrations of the sonic toothbrush. For this purpose, the polyurethane sponge was attached to the toothbrush head using a thread as shown in Supplementary Figure 2. Biofilm was removed by activating the sonic toothbrush and scrubbing the slide surface with the sponge for 40 s inside a stomacher bag (to contain splashing). The remaining steps (biofilm suspension) were performed as in the synthetic sponge technique.

### Recovery Rate Determination by Cell Enumeration

Bacterial suspensions were plated on BD Difco*™* tryptic soy agar (TSA; BD, Mississauga, Canada) after serial dilutions and incubated at 30°C for 24 h. Cell recovery was calculated using the following equation:


$${\text{Log}}_{10}\,\left(\text{CFU}/{\mathrm{cm}}^{2}\right)={\text{Log}}_{10}\,\left[\left(X/B\right)\,\left(V/A\right)\,\left(D\right)\right]$$

Where *X* is the mean number of colony-forming units (CFU); *B* is the plated volume and corresponds to 0.1 mL in the present study; *V* is the recovery buffer volume (mL); *A* is the sample surface area which is equal to 18 cm^2^ in the present study, and *D* is the dilution factor.

### Recovery Rate Determination by Optical Density

A volume of 200 μL of the biofilm suspensions collected through ultrasonication, synthetic sponge, and sonicating synthetic sponge was transferred to a microplate (96 well, Corning Incorporated, Kennebunk, ME, United States) in triplicate wells. Optical density at 600 nm (OD_600_) was measured using a Synergy H1 microplate reader (BioTek Instruments Inc., Winooski, VT, United States) managed by Gen 5 software, Version 2.07.

### Scanning Electron Microscopy

Stainless steel slides treated by the different sampling methods, toothbrush heads, and sponges were observed using a JEOL-JSM6360LV scanning electron microscope (JEOL Inc., Tokyo, Japan) at 15 kV. Prior to SEM observation, all samples were fixed overnight in an atmosphere saturated with a mixed aqueous solution of 50% glutaraldehyde and 30% formaldehyde then over-fixed in 2% osmium tetroxide solution (Electron Microscopy Sciences, Thermo Fischer Scientific, CA, United States) again overnight. Thereafter, they were mounted on aluminum stubs and were sputtered with gold. Sample preparation and microscopic imaging were carried out at the Université Laval IBIS imaging/microscopy platform.

### Statistical Analysis

All experiments were conducted in triplicate and mean data ± standard deviation was calculated for each biofilm sampling method. One-way analysis of variance (ANOVA) and Dunnett’s multiple comparison test at *p*-value ≤0.05 were performed using GraphPad Prism version 8.0.0 software for Windows (GraphPad Software, San Diego, CA, United States).

## Results

### Swabbing, Scraping, and Sonic Brushing Assessment

**FIGURE 1:**
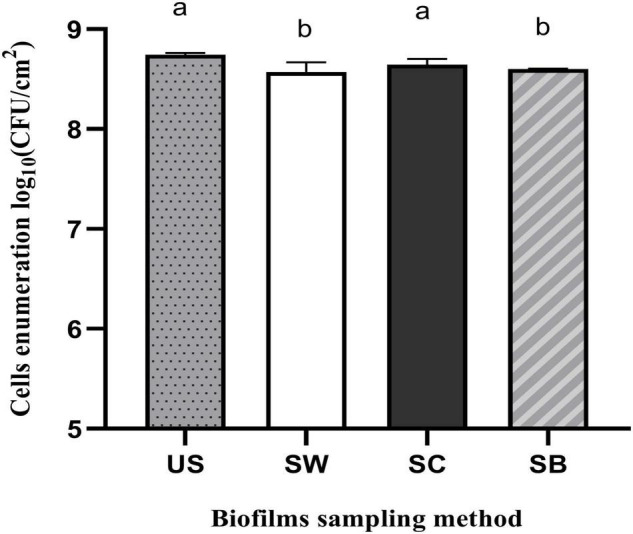
Recovery of *Pseudomonas azotoformans* PFl1A cells from biofilms grown on stainless steel slides using TSB medium by ultrasonication (US), swabbing (SW), scraping (SC), and sonic brushing (SB). Values are mean ± SD (*n* = 3). Significant differences (different letters, *p* < 0.05) are based on one-way ANOVA and Dunnett’s multiple comparison test.

The performance of three methods of biofilm sampling namely swabbing, scraping, and sonic brushing were evaluated initially after biofilm formation by *P. azotoformans* PFl1A in the CDC biofilm reactor using TSB medium. Bacterial cell recovery for each method is shown in Figure 1. Statistical analysis showed that swabbing (8.57 ± 0.10 log CFU/cm^2^) and sonic brushing (8.60 ± 0.00 log CFU/cm^2^) obtained a significantly lower (*p* = 0.016 and 0.0403, respectively) cell recovery than the standard ultrasonic method (8.74 ± 0.02 log CFU/cm^2^). In contrast, a count of 8.65 ± 0.06 log CFU/cm^2^ was achieved by using scraping, which is statistically close to the standard method (*p* = 0.1581).

**FIGURE 2:**
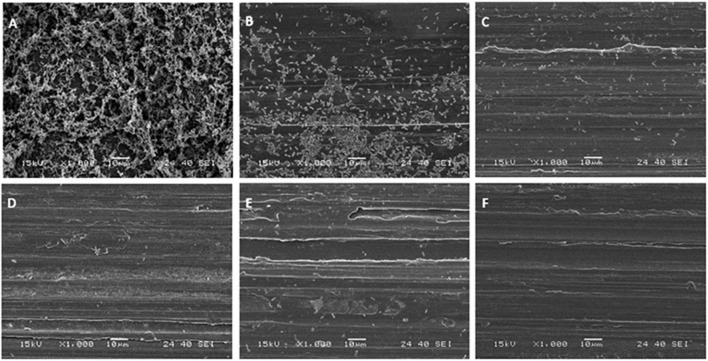
Scanning electron micrographs of *P. azotoformans* PFl1A biofilms on stainless-steel slides at 1,000× magnification after their treatment by the different sampling methods. **(A)** Untreated biofilm covered slide; **(B)** swabbing; **(C)** scraping; **(D)** sonic brushing; **(E)** ultrasonication; **(F)** biofilm-free slide (control).

**FIGURE 3:**
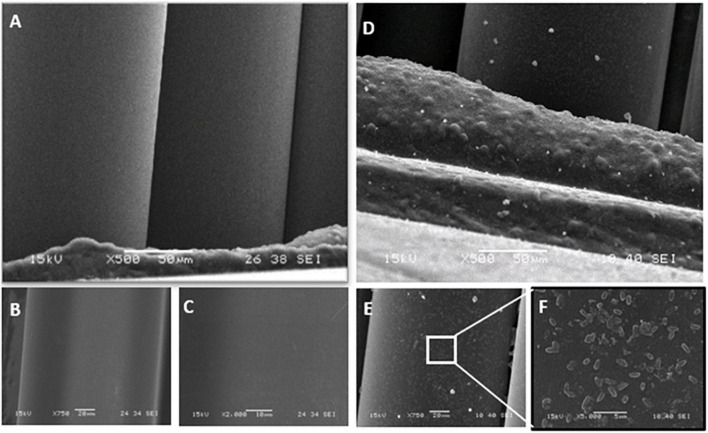
Scanning electron micrographs of the clean sonic toothbrush head bristles at 500×, 750×, and 2,000× magnification **(A–C)** and after biofilm sampling and suspending *P. azotoformans* PFl1A cells by vortex mixing and ultrasound, at 500× **(D)**, 750× **(E)**, and 5,000× **(F)**.

Stainless-steel slides were visualized by SEM before and after biofilm removal and are shown in Figure 2. As can be seen, *P. azotoformans* PFl1A formed a very dense and mature biofilm with a three-dimensional structure apparent on the positive control slide (Figure 2A). Observation revealed that a significant number of bacteria remained attached, in some areas with the biofilm structure intact, to the swabbing treated surface, which indicates incomplete biofilm recovery (Figure 2B). The remaining three sampling methods were more thorough, leaving few cells but no clusters unlike swabbing method. In these cases, a slightly higher number of cells appears to remain after scraping (Figure 2C) while the two sonic methods: sonic brushing method (Figure 2D) and ultrasonication method (Figure 2E) appeared to achieve a nearly complete removal of the biofilm from the surface. However, a few isolated bacteria could still be seen, especially at the surface grooves. In addition, SEM examination of the sonic toothbrush head shows considerable numbers of cells attached to the entire surface of the bristles and covering all the surface at their base (Figure 3). These bacteria remained attached despite the action of ultrasound and strong vortex mixing for biofilm recovery in the suspension.

### Synthetic Sponge and Sonicating Synthetic Sponge Assessment

**FIGURE 4:**
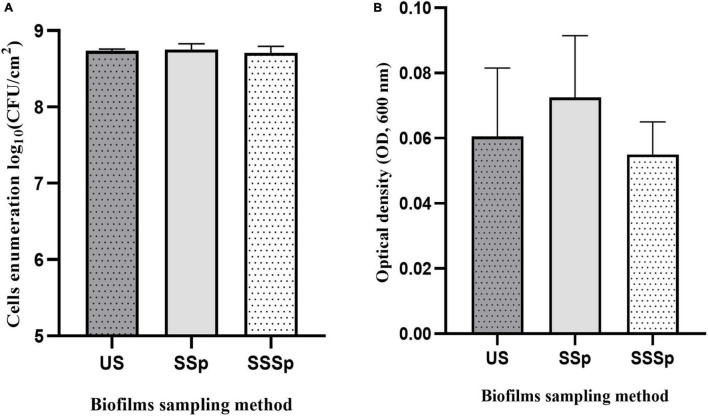
Recovery of *P. azotoformans* Pfl1A cells from biofilm grown on stainless-steel slides in TSB medium, determined by enumeration **(A)** and by measuring optical density at 600 nm **(B)**. Ultrasonication method (US); Synthetic sponge (SSp); Sonicating synthetic sponge (SSSp). Values are mean ± SD (*n* = 3). Differences are not significant (ns, *p* > 0.05), based on one-way ANOVA and Dunnett’s multiple comparison test.

To maximize both biofilm removal from the stainless-steel slides and the release of all dislodged bacteria into the recovery solutions, the use of the polyurethane sponge alone and coupled with the sonic brush were tested on *P. azotoformans* PFl1A biofilms formed under the same experimental conditions as above. The recovery rates of cells in biofilm suspension were determined by bacterial enumeration and by measuring OD_600_, and data were compared to those obtained by ultrasonication as shown in Figure 4. All these methods yielded a similar recovery of biofilms. No significant differences were found between either use of the sponge alone, the sponge coupled with the sonic brush and the standard ultrasonication method, whether the cells were counted 8.75 ± 0.08 (*p* = 0.8779), 8.71 ± 0.09 (*p* = 0.7244), and 8.74 ± 0.02 log CFU/cm^2^ (Figure 4A) or estimated by OD measurement 0.073 ± 0.019 (*p* = 0.5391), 0.055 ± 0.010 (*p* = 0.8663) and 0.061 ± 0.021 (Figure 4B), respectively, for all three techniques.

**FIGURE 5:**
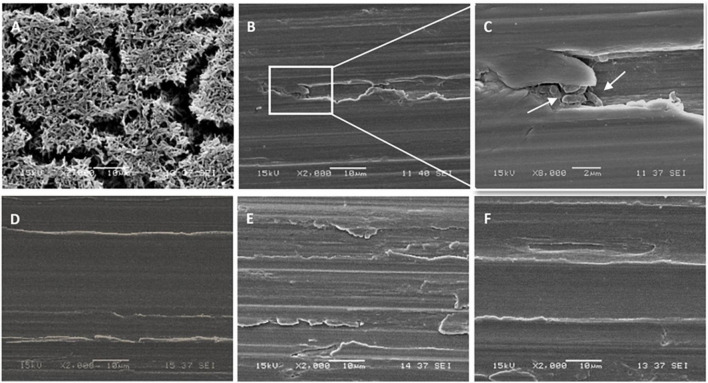
Scanning electron micrographs of the stainless-steel slides on which *P. azotoformans* PFl1A biofilm was grown then removed by different sampling methods. **(A)** Untreated biofilm covered slide; **(B)** slide surface after biofilm removal using a synthetic sponge; **(C)** portion of B at 8,000× magnification showing bacteria still lodged in a crevice (white arrows); **(D)** biofilm-free slide (control); **(E)** slide surface after biofilm removal by ultrasonication; **(F)** slide surface after biofilm removal using a synthetic sponge with sonic vibration.

SEM images of slides from which biofilms were sampled using the synthetic sponge and the sonicating synthetic sponge are presented in Figure 5. SEM micrograph of the untreated slide showed a densely growth biofilms packed with bacteria covering the entire surface and displaying a morphological characteristic of a mature biofilm (Figure 5A). When synthetic sponge method was used for sampling, the thick biofilm appeared completely removed from the surface (Figure 5B). However, at 8,000× magnification, a few cells can be seen lodged in a scratch on the stainless-steel slide (Figure 5C). Furthermore, no biofilm remained on the slides after ultrasonic treatment (Figure 5E) or use of the sonicating synthetic sponge method (Figure 5F), not even in scratches.

**FIGURE 6:**
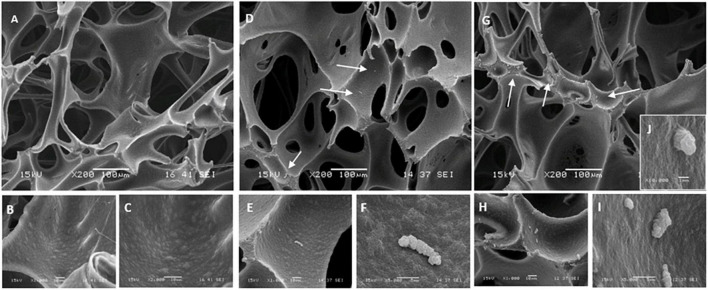
Scanning electron micrographs of the polyurethane sponge with *P. azotoformans* PFl1A biofilm remaining after sampling. **(A–C)** Clean sponge observed at 200×, 1,000×, and 2,000× magnification. **(D–F)** Images of the sponge at 200×, 1,000×, and 5,000× magnification, after biofilm removing and 30 s of intense agitation with the Pulsifier to suspend sampled cells. **(G–J)** Images of specify the sonicating sponge at 200×, 1,000×, 5,000×, and 10,000× magnification, after biofilm removing and 30 s of intense agitation with the Pulsifier. White arrows show remnants of *P. azotoformans* PFl1A biofilm.

Visualization by SEM of sponges (synthetic sponge and sonicating synthetic sponge) after sampling also provided an indication of the amount of biofilm that remained clinging to these sampling devices (Figure 6). Remnants of the *P. azotoformans* PFl1A biofilm were seen distributed sparsely (Figures 6D,G, white arrows) whether the sponge was associated with the sonic toothbrush or not. At higher magnifications (1,000× in Figures 6E,H, 5,000× in Figures 6F,I), such biofilm appeared as a gelatinous deposit, likely a fragment of polymer matrix. Contrary to the sonic brush head, bacteria were not spread on the sponge surface and no clusters were observed. The highly magnified image (10,000×) of one of these fragments revealed that these deposits did not contain bacteria, thus confirming that the residues of biofilms left on the synthetic sponges were lack of bacterial cells (Figure 6J).

### Efficiency of Sampling Biofilms Grown in Skimmed Milk

**FIGURE 7:**
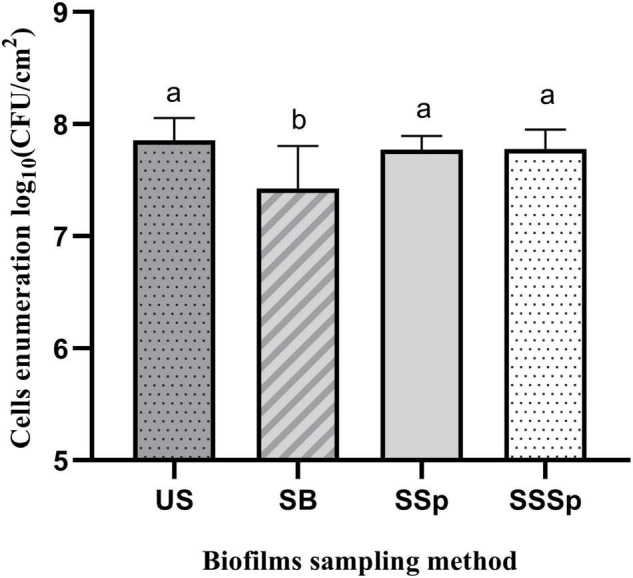
Recovery of *P. azotoformans* Pfl1A cells from biofilm grown on stainless-steel slides in sterile skim milk, by ultrasonicating (US), sonic brushing (SB), synthetic sponge (SSp), sonicating synthetic sponge (SSSp). Values are mean ± SD (*n* = 3). Significant differences (different letters, *p <* 0.05) are based on one-way ANOVA and Dunnett’s multiple comparison test.

The sampling methods that detached the most biofilm, based on cell counts or on SEM images, were tested on *P. azotoformans* PFl1A biofilm formed in sterile skim milk. Bacterial enumerations in suspensions obtained by sonic brushing, synthetic sponge, and sonicating synthetic sponge are shown in Figure 7. Cell densities in these biofilms were lower than in those grown in TSB medium, never exceeding 7.86 ± 0.20 log CFU/cm^2^ that was reached by the ultrasonication standard method. No significant differences between synthetic sponge (7.77 ± 0.12 log CFU/cm^2^), sonicating synthetic sponge (7.78 ± 0.17 log CFU/cm^2^) and ultrasonication were observed (*p* = 0.8694 and 0.8990, respectively). However, biofilm recovery by sonic brushing was statistically significant (*p* = 0.014) compared to the other methods and demonstrated the lowest bacterial count (7.43 ± 0.38 log CFU/cm^2^).

## Discussion

The polymer matrix produced by microorganisms within biofilms ensures tenacious attachment of cells to surfaces such as stainless steel, making their recovery more challenging, especially when they are grown under dynamic flow conditions. Currently, no biofilm sampling method in common use in dairy processing plants provides a complete removal from the surface area tested. Ultrasound, on which some laboratory methods are based, namely the ASTM standard (ASTM International, 2019), can easily disrupt the matrix structure of biofilm and release attached microorganisms using the sonicating bath. However, this approach cannot be considered in an industrial application for in-plant sampling. The aim of this study was to identify a sampling method that could be as effective and reliable as ultrasonication, as well as suitable for routine use in industrial settings.

Among the methods compared, swabbing was the least effective in terms of biofilm removal from surfaces. Based on cell counts and direct observation by SEM, dislodging of biofilms from the stainless-steel slide was incomplete. The findings are corroborated with the studies previously reported, where swabbing has been found less effective to detach the adhering bacteria due to the insufficient pressure applied during rubbing of the test surface (Yamaguchi et al., 2003; Moore and Griffith, 2007) and to the difficulty of transferring bacteria into the analytical suspension (Moore and Griffith, 2007). In another study, Asséré et al. (2008) observed that the recovery of *Pseudomonas fluorescens* and *Salmonella typhimurium* from biofilm grown on polyvinyl chloride surfaces was poor using swabs compared to ultrasound at different frequencies. The low efficiency of the swab technique has been attributed to high capillary action and absorbency of the cotton and its ability of retaining bacterial cells due to tightly entangled structure of the fibrous structure (Keeratipibul et al., 2017). Furthermore, it has been reported that *Listeria monocytogenes* biofilm can remain entrapped within the swab fibers following sampling, even after vigorous vortex agitating for 20 s (Faille et al., 2020).

Similarly, a lower recovery rate was observed by sonic brushing method compared to ultrasonication in the present study. However, a more complete biofilm removal was obtained than the swabbing method as confirmed by SEM examination. This finding suggests that sonic brushing could effectively dislodge the biofilms. This is supported by several earlier previous studies conducted on dental biofilm removal using the sonic toothbrush (Heersink et al., 2003; Singh et al., 2011; Re et al., 2015). It has been demonstrated that the detachment of biofilm by sonic brushing ascribed to the hydrodynamic activity resulting from both the toothbrush head vibration and sound pressure waves. In this “non-contact” brushing, air microbubbles are generated due to the high-speed oscillating action caused by the bristle motion in the fluid. Vigorously injected into the biofilm, they collide with the adherent bacteria (Sharma et al., 2005). Such effect can be enhanced by acoustic energy transfer, which triggers the expansion of the biofilm followed by deformation and ultimately disruption, depending on the amount of absorbed energy (Busscher et al., 2010).

An earlier study conducted by Mclnnes et al. (1993) revealed that low-frequency (200 Hz) acoustic energy provided either by a laboratory acoustic generator or a Sonicare^®^ toothbrush cause significant damage to the fimbriae of the oral bacterial species *Actinomyces viscosus* after 30–60 s of exposure. This frequency was found to promote removal of bacteria from the test surface and to break up cell clusters. The sonic brushing treatment in the present study was for 40 s at 257 Hz, which should have been sufficient for biofilm detachment of *P. azotoformans* PFl1A. However, even though sonic brushing successfully performed dislodging the biofilm, bacterial counts recovered in the analytical suspension were lower than expected. SEM imaging revealed a significant bacterial charge remaining in the toothbrush head, where biofilm cells were clearly trapped on bristles, between them and at their base, despite ultrasonication and vortex agitation. In fact, the bristles are made of nylon, a polymer material well-known to absorb water (Shakiba et al., 2021), which might be preventing the cells to be transferred into the sampling suspension (Black et al., 2007; Park et al., 2014). In addition, their bundling (closely juxtaposed) helps them retain many of the cells freed from the biofilm (Efstratiou et al., 2007).

Since previous studies suggested that bacteria are easier to release from polyurethane surfaces (Pearce and Bolton, 2005; Zwirzitz et al., 2020), a method combining sonication with a polyurethane sponge instead of using the bristle head was tested in the present study to maximize the recovery of detached biofilm cells. The cell recovery rate was not significantly different using synthetic sponge alone and sonicating synthetic sponge methods since both achieved a similar cell recovery to that of standard ultrasonication procedure. The efficiency of the polyurethane sponge to remove biofilms is attributed to the abrasiveness of the material, which is enhanced by the high-speed motion of the toothbrush head. Furthermore, SEM analysis showed a porous structure with large cavities in the synthetic sponges, allowing bacteria to be transferred easily to the biofilm solution, unlike the aligned arrangement of the tightly grouped toothbrush bristles. Interestingly, no residual bacteria, either in single cells or clusters, were observed on sponges used for biofilm sampling after vigorous agitation in the Pulsifier^®^. Nevertheless, some biofilm-derived matter, possibly residues of the EPS matrix, was visible on the surface of the sponges. Based on observation at magnifications of 8,000× and 10,000×, it has been confirmed that this residual matter was not cellular, since *P. azotoformans* cells were clearly seen on Sonicare^®^ bristles at 5,000×. It is likely that some physicochemical characteristic such as the water-repelling property of polyurethane makes this material refractory to bacterial adherence and retention.

SEM analysis demonstrated the presence of a residual cluster of biofilms within the crevice on the slide treated by synthetic sponge method. However, combining the sponge method with sonication allowed complete detachment of the biofilm from the stainless-steel surface, even from the scratches. This result was comparable to that obtained by sonic brushing. In both cases, the vibrating frequency was 257 Hz (assuming that replacement of the bristles with the sponge did not change the frequency). This finding suggests that sonic vibration may play a crucial role in dislodging adherent cells, especially on surfaces with cracks and crevices, in which bacteria lodge and grow to form biofilms. In this instance, sonication may break up the EPS matrix, disrupt the biofilm and thereby release entrapped bacteria from spaces that the sponge alone cannot reach. These data are consistent with a study on *Streptococcus mutans* oral biofilm removal using a Sonicare^®^ toothbrush at 260 Hz. The effectiveness of this device without direct contact with the surface has been shown, suggesting its action at short distance on areas that bristles cannot access (Heersink et al., 2003).

To mimic the conditions of biofilm formation in dairy plants wherein milk is in prolonged contact with the stainless-steel insides of processing equipment, the experiment was carried out under the same dynamic conditions by replacing the TSB medium with sterile skim milk. Also, it is noteworthy that the bacterial strain used in the present study was isolated from a dairy processing plant, where it causes discoloration spoilage of milk. Interestingly, we found that biofilm density in the CDC reactor fed by skim milk was 1log CFU/cm^2^ lower than the one grown in the TSB (100 mg/L). Considering that skim milk has the much higher nutrient content, especially in terms of proteins, the stainless-steel surfaces covered by the conditioning film become saturated with these adsorbed macromolecules. In fact, milk proteins do coat the surface and consequently, they may have interfered with bacterial adhesion without necessarily having to prevent formation of biofilms or affect its strength. Previous studies have reported that α-casein, β-casein, κ-casein, α-lactalbumin, and serum albumin can lessen cell attachment on stainless-steel surfaces (Helke et al., 1993; Barnes et al., 2001; Parkar et al., 2001). The layer of adsorbed proteins could weaken the interactions between cell wall and stainless-steel surface by disrupting some of the physico-chemical bonding involved in the initial stage of biofilm formation.

Efficiency assessment of sampling methods on biofilm formed in milk demonstrated no significant differences between the number of bacterial cells recovered by the synthetic sponge, the sponge with sonic vibration and the ASTM standard ultrasonication method, whereas sonic brushing was noticeably less efficient, likely because of losses of bacterial cells due to the mechanisms discussed above. This finding suggests that milk constituents had no impact on biofilm sampling. Any involvement of protein accumulation and other component of milk, though affecting biofilm density by reducing bacterial attachment to the surface, does not compromise biofilm removal in the conditions used in this study. The polyurethane sponge method, with or without sonic vibration, ensures optimal sampling of the biofilm, comparable to that obtained by the standard ultrasonication method.

In conclusion, this work expands the information available on biofilm formation, using a representative biofilm-forming species, namely *P. azotoformans* PFl1A grown under dynamic conditions like those that characterize dairy processing. In addition, this study introduces a suitable method for sampling biofilms adequately. It thus has been demonstrated clearly that a polyurethane sponge used with or without sonic vibration can be used to detach and recover practically all sessile cells, whereas a complementary and a synergistic effect was ensured by sonic vibration on surfaces with imperfections such as crevices. The equipment needed for the sonic synthetic sponge method is easy to transport and use and is adaptable to cramped spaces, making it a promising potential alternative for on-site routine sampling of biofilms. Further investigation should be undertaken to validate the performance of these approaches with other food matrices such as poultry, vegetables, or seafood, since biofilms are problematic also in facilities processing these foods. This would allow generalizing of their use in the sampling of biofilms throughout the food industry.

## Data Availability Statement

The original contributions presented in this study are included in the article/Supplementary Material, further inquiries can be directed to the corresponding author.

## Author Contributions

NN conducted the experiments and prepared the manuscript. CG provided scientific advice. LS and JF were involved in some assays. NN, CG, IF, SL, and JJ designed the study and reviewed the manuscript. JJ supervised the project. All authors contributed to the article and approved the submitted version.

## Conflict of Interest

The authors declare that the research was conducted in the absence of any commercial or financial relationships that could be construed as a potential conflict of interest.

## Publisher’s Note

All claims expressed in this article are solely those of the authors and do not necessarily represent those of their affiliated organizations, or those of the publisher, the editors and the reviewers. Any product that may be evaluated in this article, or claim that may be made by its manufacturer, is not guaranteed or endorsed by the publisher.
