# Supplementary figures and images for: Comparative Study of Different Sampling Methods of Biofilm Formed on Stainless-Steel Surfaces in a CDC Biofilm Reactor
Source: Front Microbiol. 2022 Jun 13;13:892181. doi: 10.3389/fmicb.2022.892181 (PMC9234490; doi:10.3389/fmicb.2022.892181)

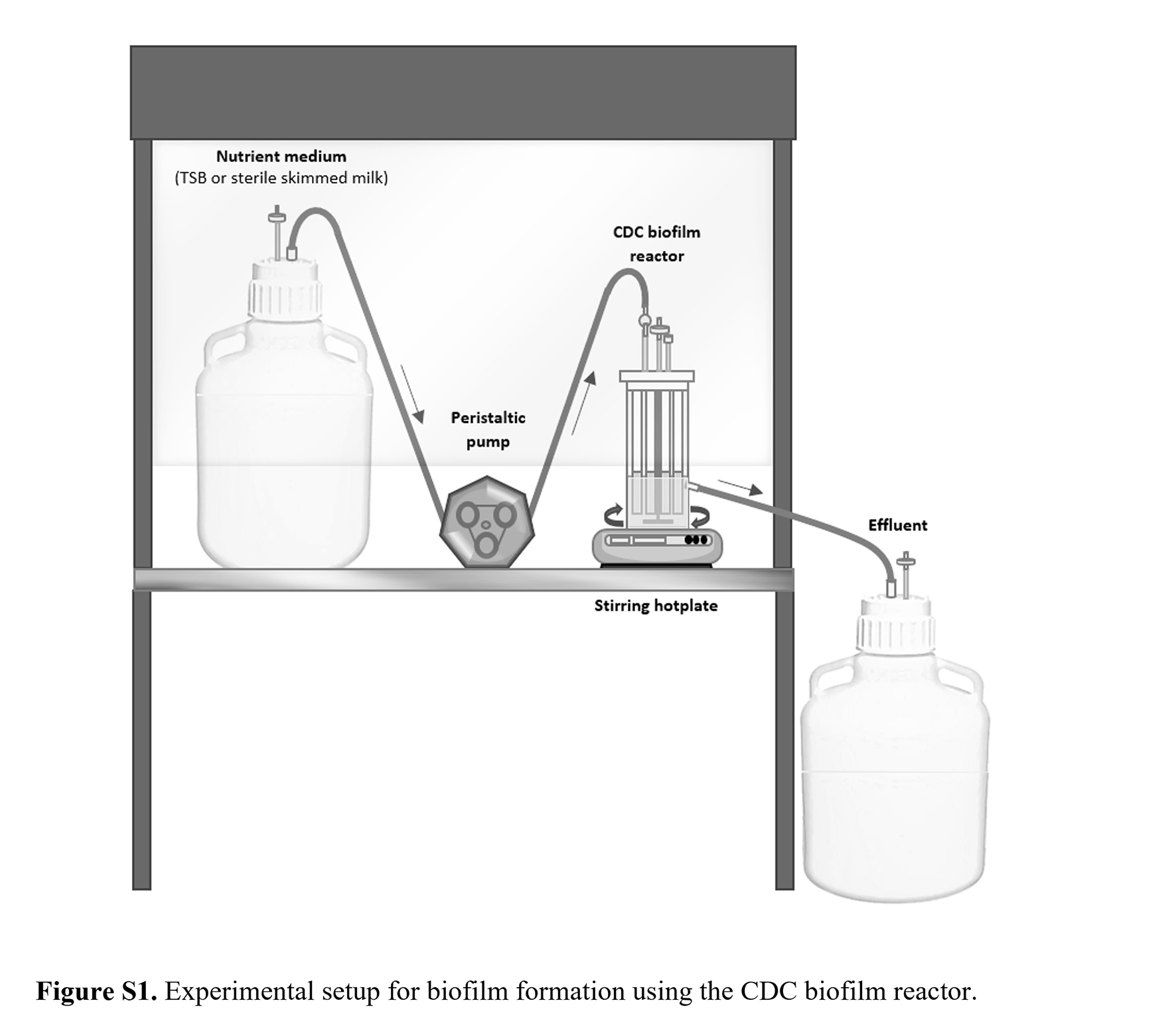

Supplement: Supplementary file 1 [file Image_1.TIFF]

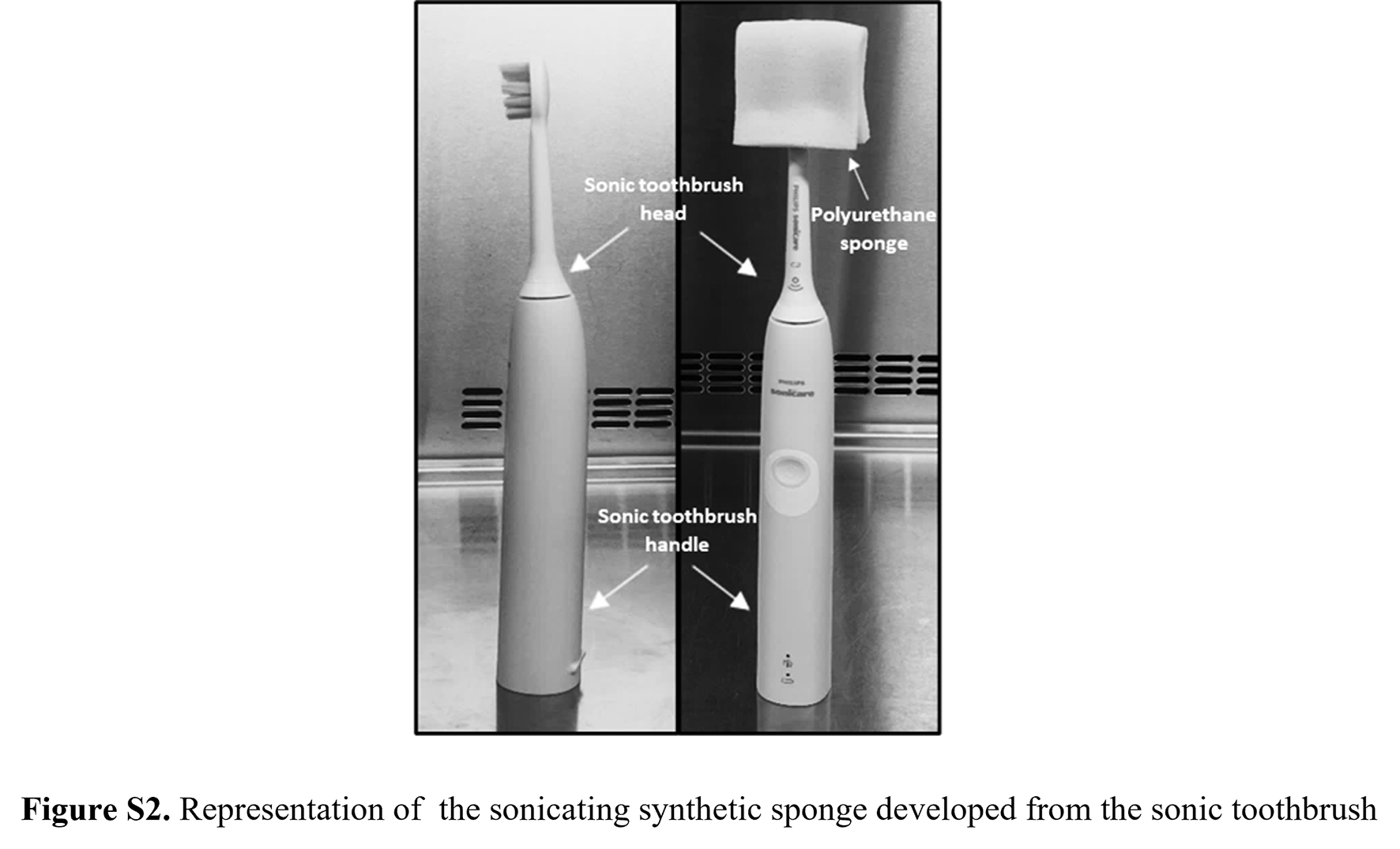

Supplement: Supplementary file 2 [file Image_2.TIFF]
